# Tumor Infiltrating Lymphocytes in Pet Rabbit Mammary Carcinomas: A Study with Relevance to Comparative Pathology

**DOI:** 10.3390/ani10081437

**Published:** 2020-08-17

**Authors:** Sandra Schöniger, Sophie Degner, Qian Zhang, Claudia Schandelmaier, Heike Aupperle-Lellbach, Bharat Jasani, Heinz-Adolf Schoon

**Affiliations:** 1 Targos Molecular Pathology GmbH, Germaniastrasse 7, 34119 Kassel, Germany; bharat.jasani@targos-gmbh.de; 2 Institute of Veterinary Pathology, University of Leipzig, An den Tierkliniken, 04109 Leipzig, Germany; soraehse@web.de (S.D.); schoon@vetmed.uni-leipzig.de (H.-A.S.); 3 Institute of Anatomy, Experimental Neurobiology, Goethe-University, Theodor-Stern-Kai 7, 60590 Frankfurt/Main, Germany; q.zhang@em.uni-frankfurt.de; 4 Laboklin GmbH & Co. KG, Steubenstrasse 4, 97688 Bad Kissingen, Germany; schandelmaier@laboklin.com (C.S.); aupperle@laboklin.com (H.A.-L.)

**Keywords:** animal model, biomarker, breast cancer, comparative pathology, mammary carcinomas, light microscopy, *Oryctolagus cuniculus*, pet rabbit, tumor infiltrating lymphocytes, translational medicine

## Abstract

**Simple Summary:**

The interaction between tumors and immune cells influences tumor fate, i.e., regression, growth, or even metastases. The evaluation of tumor infiltrating lymphocytes (TILs) in human breast cancer has prognostic value. Pet rabbits develop spontaneous mammary carcinomas and have an immune system that is comparable with that of humans, so that they have the potential to provide an animal model for human breast cancer. To further substantiate this similarity, this study examined TILs in 107 pet rabbit mammary carcinomas according to criteria established for human breast cancer. For TIL evaluation routinely stained microscopic sections were examined by light microscopy. Relevant histological and immunohistochemical tumor characteristics were obtained from a data base. Results showed that increased presence of stromal TILs was statistically associated with histological tumor features indicative of a less aggressive biological behavior, i.e., reduced tumor cell proliferation and a lower histological grade. The expression by tumor cells of calponin, a presumed tumor suppressor protein, was also associated with their reduced proliferation and a higher percentage of stromal TILs. Data suggest that higher percentages of stromal TILs may have the potential to serve as favorable prognostic indicator in rabbit mammary carcinomas and support the value of pet rabbits for comparative research.

**Abstract:**

Tumor infiltrating lymphocytes (TILs) serve as prognostic biomarker in human breast cancer. Rabbits have the potential to act as animal model for human breast cancer, and close similarities exist between the rabbit and human immune system. The aim of this study is to characterize TILs in pet rabbit mammary carcinomas and to statistically correlate results with histological and immunohistochemical tumor characteristics. Microscopic evaluation of TILs was performed in hematoxylin and eosin stained sections of 107 rabbit mammary carcinomas according to international guidelines for human breast cancer. Data on histological features of malignancy, estrogen and progesterone receptor status and calponin expression were obtained from the data base. This study revealed a statistical association between stromal TILs in the central tumor (CT) and infiltrative margin. Higher maximal percentages of stromal TILs at the CT were statistically correlated with decreased mitotic count and lower tumor grade. An increased number of calponin positive tumor cells was statistically associated with a lower mitotic count and a higher percentage of stromal TILs. Results suggest that higher percentages of stromal TILs are useful biomarkers that may point toward a favorable prognosis in rabbit mammary carcinomas and support the concept of the use of rabbits for translational research.

## 1. Introduction

Pet rabbits serve as excellent animal models for numerous infectious, degenerative, and neoplastic human diseases [1,2,3,4,5,6,7,8]. This includes the investigation of disease-associated molecular and cellular mechanisms as well as the development of vaccines and treatment options [4,7,8]. The suitability of rabbits as animal models for human diseases is facilitated by the availability of comprehensive data on their innate and adaptive immune mechanisms [1,8,9], close similarities between the rabbit and human immune systems [1,4], and very similar expression and functions of key genes between humans and rabbits [2]. Recent investigations on spontaneous mammary carcinomas of pet rabbits, identified the pet rabbit as potential animal model for certain types of spontaneous human mammary tumors [10,11,12]. This eligibility is further supported by the following aspects: In contrast to rodents, mammary glands of does and women contain independent ductal systems with separate teat orifices [13]. The rabbit size allows the application of diagnostic and therapeutic procedures that are also used in humans [13]. The age expectancy of rabbits is approximately 6–13 years [14], and thus will permit enough follow-up time for tumor recurrence, metastases and survival time.

It has been shown that most rabbit mammary tumors are carcinomas, i.e., 50–98% depending on the respective study, the majority of these being adenocarcinomas [10,11,12,15,16]. Recent investigations on rabbit mammary carcinomas analyzed in detail the histological features [10,15,16], estrogen and progesterone receptor status [10], as well as the intra-tumoral presence of retained non-neoplastic myoepithelial cells and tumor cells with a myoepithelial differentiation [11,15]. Definitive prognostic factors are not available yet, and currently the only therapeutic option is surgical excision [12].

The immune system plays a major role in the body′s defense against tumor development and progression [17,18,19]. Tumor infiltrating lymphocytes (TILs) can be found in the central tumor, the invasive margin, and the peritumoral stroma [17,20,21,22] and include different lymphocyte subpopulations such as cytotoxic T cells (Tc1, Tc2), T helper cells (Th1, Th2), Th17 cells, regulatory T cells (T regs), B cells, and plasma cells [17,20,21,22].

CD8+Tc1 [17,19,21,23] and CD4+Th1 cells [17,19] are the main mediators of adaptive anti-tumor immune responses. A high density of Th1 cells and Tc1 in the tumor area represents a favorable prognostic factor in several types of cancer [17] including certain types of breast cancer [20]. T regs promote tumor progression by acting immunosuppressive [19,21]. Studies revealed divergent roles of B cells [17,19,24], Th2 cells [17,21,25], as well as Th17 cells [17,20,21]. The respective functions of the latter cell populations are likely influenced by the tumor type and molecular subtype [17], stage of disease [17], as well as differences of the tumor microenvironment [17,21,24].

Notably, despite the phenotypic and functional heterogeneity of TILs, the general assessment of TILs in routinely stained (hematoxylin eosin stained) tissue sections has prognostic significance for certain types of human breast cancer and other malignant tumors [20,21,26]. In certain types of breast cancer, a favorable prognostic effect of increased stromal TILs is reported, e.g., triple-negative tumors [21,27,28], estrogen receptor (ERα) negative cancer [29], and HER2 positive malignancies [21,30].

For a standardized evaluation of TILs in human breast cancer and other solid tumors, international guidelines exist [20,21,22,31]. The aim of this investigation was to examine TILs according to these guidelines in pet rabbit mammary carcinomas and to correlate obtained results with histological features, ERα and progesterone receptor (PR) status, as well as calponin-expression in tumor cells, to check their possible prognostic significance.

## 2. Materials and Methods

### 2.1. Animals and Tissue Samples

This retrospective study was performed on archived formalin-fixed paraffin-embedded tissue (FFPE) samples of 107 mammary carcinomas from 107 pet rabbits, respectively. Immediately after their excision in veterinary practices, tissue samples had been fixed in 10% buffered formalin for 24 to 72 h. After their arrival in the diagnostic laboratory, tissue samples were trimmed, routinely processed, and embedded in paraffin-wax.

The age of the rabbits was reported for 97 animals and ranged from 1.5 to 10 years with a mean age of 5.3 years and a standard deviation (SD) of 1.6 years. The sex was known for 97 animals; of these 78% (*n* = 76) were female and 22% (*n* = 21) female spayed. Regarding the rabbits with known breed (*n* = 52), there were 31 dwarf rabbits, 7 dwarf lop rabbits, 5 lop rabbits, 5 lion head rabbits, 1 Teutoburger rabbit, 1 rex rabbit, 1 angora rabbit, and 1 lop mixed breed rabbit.

Hematoxylin eosin (HE) stained tissue section of the 107 rabbit mammary carcinomas were obtained from the database and re-examined to confirm the presence of a mammary adenocarcinoma. These tissue samples were also used for the microscopic evaluation of TILs. In addition, histological features associated with the degree of tumor differentiation (reported below), the ERα and PR status as well was the percentages of calponin positive tumor cells were extracted from the databases and diagnostic records [10,11].

### 2.2. Light Microscopic Evaluation of Tumor Infiltrating Lymphocytes

For each of the rabbit mammary carcinomas, TILs were evaluated according to the international guidelines for human mammary carcinomas [20,21] using a Zeiss microscope scope A1 with an ocular field number of 23. As defined by Salgado et al. [20] and Hendry et al. [21], TILs represent stromal and intra-tumoral lymphocytes and plasma cells. Stromal TILs are defined as percentage of stroma area occupied by TILs over the total stromal area [20,21]. Intra-tumoral TILs (IT TILs) represent the epithelial tumor area covered by TILs in relation to the entire epithelial tumor tissue [20,21]. They are defined as TILs associated with tumor cell nests that have direct cell to cell contact with cancer cells with no intervening stroma [20,21]. TILs were evaluated separately within the central tumor (CT) and the invasive margin (IM) [20,21]. The IM is defined as 1-mm wide zone at the tumor periphery centered at the delineation of the outer margin of the tumor cell nests [21]; it is composed of an inner rim of tumor tissue (500 µm) and an outer rim of peritumoral stroma (500 µm) [21]. TILs were analyzed with the 20× objective, and IT TILs were confirmed as TILs using the 40× objective. From these data, the average percentage of TILs over the entire stromal or intra-tumoral area in the CT and IM, as well as maximal and minimal percentages of TILs per 20× objective field of view (20× objective FOV) were obtained. Within the fibrous connective tissue of the adjacent non-neoplastic mammary tissue, lymphocytes and plasma cells were determined as well by using the 20× objective FOVs. Percentages of TILs < 10% were reported in 1% intervals and those ≥ 10% in 5% intervals.

### 2.3. Data Obtained from Diagnostic Records

Histological parameters extracted from the databases were the tumor area with a tubular growth pattern in percent of the entire tumor area, the mitotic count per ten 40× high power fields (HPFs) under consideration of the field number of the microscope, the degree of nuclear pleomorphism, as well as the degree of tumor invasion [10,11,15]. The results of the former three parameters were used to determine the histological tumor grade [32,33,34]. Depending on the degree of tissue infiltration, tumors were classified as those with mild, moderate, or marked invasive behavior [10,11,15]. Necrosis was recorded as minimal, mild, moderate, and marked, if it affected <10%, 10–39%, 40–69%, and ≥70% of the tumor area, respectively [10].

Immunohistochemical data obtained from the diagnostic records were percentages of ERα and PR positive tumor cells, the immunoreactive score (IRS), the histological score (H-score), as well as the percentage of calponin positive tumor cells [10,11]. Carcinomas had been immunostained for ERα, PR, and calponin with the peroxidase anti-peroxidase (PAP) method and 3,3′-diaminobenzidin as chromogen by using the following primary cross-reactive antibodies: mouse anti-human ERα (clone 6F11, Novocastra Laboratories, Newcastle upon Tyne, UK: 1:20 diluted), mouse anti-human PR (clones 16 and SAN 27, Novocastra Laboratories: 1:100 diluted), and mouse anti-human calponin 1 (clones SPM 169, Zytomed Berlin, Germany: 1:200 diluted) [10,11]. By image analysis, the percentages of tumor cells positive for ERα, PR, and calponin had been determined [10,11]. ERα and PR positive cells had been further subclassified in those with a mild (1+), moderate (2+), or strong (3+) immunoreaction and for each tumor the IRS [35] and the H-score [36] had been calculated according to the formulas provided below [10].

$$IRS=\;\frac{\left(1+\;cells\right)+\left(2+\;cells\;\times \;5\right)+\left(3+\;cells\;\times \;10\right)}{100};\;H-score\;=\;(1+\;cells)\;+\;(2+\;cells\;\times \;2)\;+\;(3+\;cells\;\times \;3)$$

### 2.4. Statistical Evaluation

The statistical analysis was done by using IBM SPSS software version 25 (IBM SPSS Inc., Armonk, NY, USA). Data are presented as mean ± SD. The correlation between two investigated factors was analyzed with Pearson’s co-relation coefficient, and the significance threshold was set at 0.05.

## 3. Results

### 3.1. Stromal Tumor Infiltrating Lymphocytes

**Figure 1:**
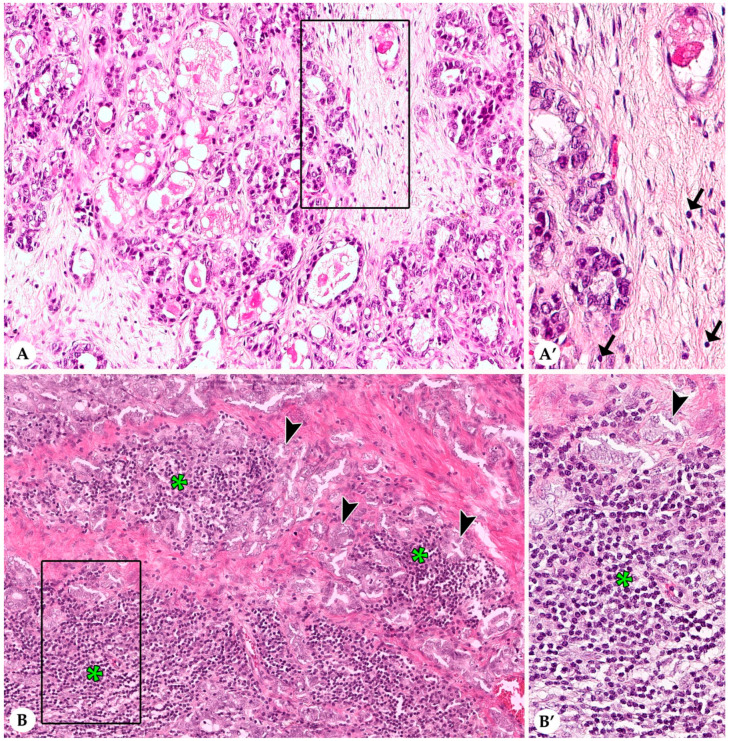
Stromal tumor infiltrating lymphocytes (TILs) within the central tumor (CT) of pet rabbit mammary carcinomas. Illustrated are two representative tumor areas with very few stromal TILs (**A**,**A’**) and multifocal aggregates of stromal TILs (**B**,**B’**), respectively. (**A**,**A’**) Grade I carcinoma with a tubular growth pattern and moderate secretory activity. Occasional stromal TILs (less than 1%) are present in the 20× objective field of view (FOV) shown in A. The rectangular area delineated in A is illustrated in A’ in higher magnification. Individual rare stromal TILs are labelled by arrows. (**B**,**B’**) Stromal TILs (asterisks) form multifocal aggregates that dissect between tubular structures lined by tumor cells (arrowheads). The 20× objective FOV present in B contains an estimate of 50% stromal TILs. The area delineated by a rectangle is shown in B’ in higher magnification.

**Figure 2:**
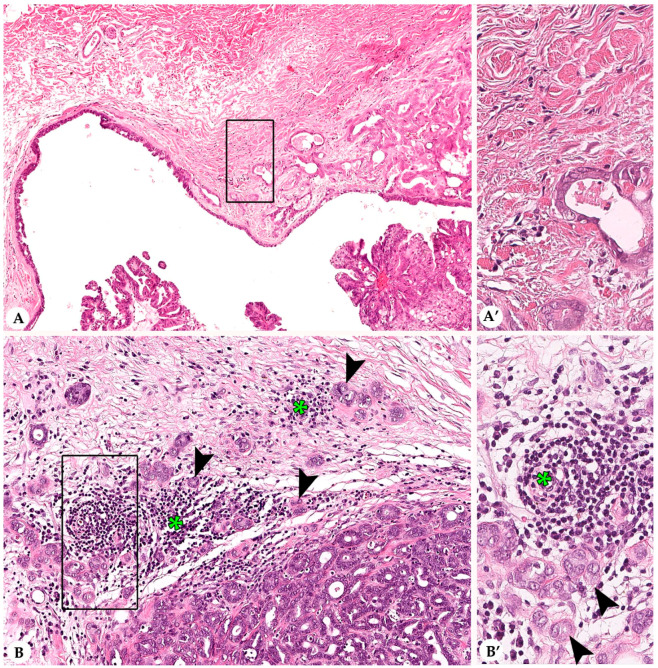
Stromal tumor infiltrating lymphocytes (TILs) within the infiltrative margin (IM) of pet rabbit mammary carcinomas. Shown are two representative tumors with nearly complete absence of stromal TILs (**A**,**A’**) and multifocal clusters of stromal TILs (**B**,**B’**) at the IM, respectively. (**A**,**A’**) Grade II carcinoma with tubular and cystic growth patterns and mild secretory activity. The IM contains no unequivocal stromal TILs. In A, the 10× objective field of view (FOV) is depicted. The rectangle in A delineates the area that is shown in A’ in higher magnification. (**B**,**B’**) Grade II carcinoma with a predominantly tubular growth. TILs (green asterisks) are mostly located between and adjacent to the infiltrative tumor cell nests (arrowheads). In B, the 20× objective FOV is shown and contains approximately 25% stromal TILs. The area that is contained within the rectangle is depicted in B’ in close up.

In most rabbit mammary carcinomas average stromal TILs encompassed up to 10% in the TC and at the IM. More than 50% average stromal TILs, a feature of human lymphocyte-dominant breast cancer [20,21], were observed in two tumors in the CT and in none of the tumors at the IM. Results are summarized in Table 1 and described in detail below. Figure 1 and Figure 2 show representative images from cases with low and high stromal TILs in the CT and at the IM, respectively.

In the CT, average stromal TILs ranged from 0–70% with a mean value (MV) of 6% and a standard deviation (SD) of 12%. Majority of carcinomas (89%; 95/107) had 0–10% average stromal TILs, whereas 9% (10/107) and 2% of carcinomas (2/107) contained an average of 11–50% and >50% stromal TILs, respectively. Maximal percentages of stromal TILs per 20× objective FOV varied between 1 and 90% (MV: 19%; SD: 23%). Minimal percentages of average stromal TILs per 20× objective FOV showed a range from 0–60% (MV: 2%; SD: 7%). The distribution of stromal TILs within the TC was often heterogenous with a mean difference of 17% (SD: 21%) between the maximal and minimal percentages of TILs per 20× objective FOV. In 59% of tumors (63/107) this difference ranged from 1–10%, whereas in 35% (37/107) and 6% (7/107) of the carcinomas it varied between 11–50% and >50%, respectively.

An IM was present in 102/107 (95%) sections of rabbit mammary carcinomas, whereas in four cases due to incomplete excision of the tumor tissue an IM could not be evaluated and in one case the relatively small size of the tumor did not allow a separate evaluation of CT and IM. Average stromal TILs at the IM ranged from 0–30% (MV: 3%; SD: 5%). In 95% of the tumors, average stromal TILs at the IM were ≤10%. The remaining carcinomas contained average stromal TILs of 20% (3/102) and 30% (2/102) TILs at the IM, respectively. Per 20× objective FOV at the IM, maximal percentages of stromal TILs showed a range from 0–70% (MV: 10%; SD: 13%) and minimal percentages extended from 0–10% (MV: 1%; SD: 2%). Although in some tumors stromal TILs at the IM formed hot spots, the overall distribution of stromal TILs at the IM was less heterogenous than in the CT, i.e., the mean difference between the maximal and minimal percentages of TIL per 20× objective FOV was 9% (SD: 13%). In detail, in 78% (80/102) of the carcinomas this difference ranged from 1–10%, while in 20% (20/102) and in 2% (2/102) of these tumors it varied between 11–50% and >50%, respectively. Notably, at the IM, stromal TILs were mostly located within the 500-µm wide inner rim of the IM compared to the 500-µm wide outer rim of the IM.

### 3.2. Infiltrating Lymphocytes within Nests of Carcinoma Cells

**Figure 3:**
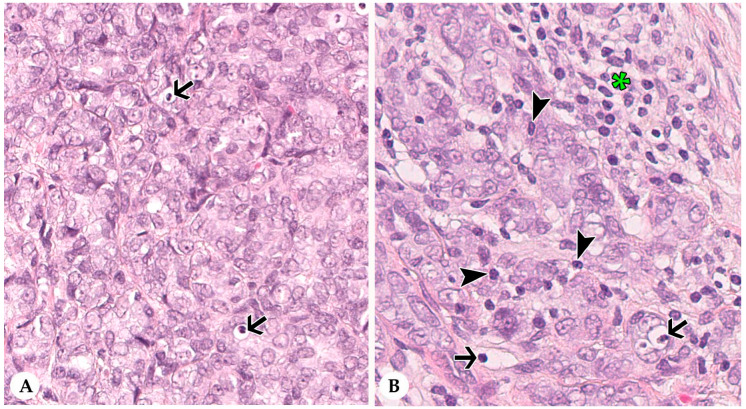
Intra-tumoral infiltrating lymphocytes (IT TILs) of pet rabbit mammary carcinomas. Depicted are two representative tumors with almost lack of IT TILs (**A**) and some IT TILs (**B**), respectively. In comparison to stromal TILs (asterisk), IT TILs (arrowheads) are immediately associated with tumor cells (arrowheads). Intra-tumoral cell fragments (arrows) are present (**A**,**B**).

IT TILs were formed solely by lymphocytes (Figure 3). Results are summarized in Table 1. In the CT, the average percentage of IT TILs varied between 0–5% with MV of 0.24% and SD of 0.67%. Carcinomas with an average of 0% IT TILs dominated (82%; 88/107), whereas 1% average IT TILs occurred in 15% of the carcinomas (16/107). One tumor each contained average IT TILs of 2%, 3%, and 5%, respectively. Minimal numbers of IT TILs per 20× objective FOV ranged from 0–1% (MV: 0.06%; SD: 0.23%) and maximal numbers from 0–25% (MV: 0.98%; SD: 3%).

At the IM, average percentages of IT TILs differed between 0% and 10% (MV: 0.24%; SD: 1%). Most tumors (87%; 89/102) contained 0% average IT TILs, whereas 9% (9/102) and 3% (3/102) of carcinomas had an average of 1% and 2% IT TILs. In one tumor, 10% average IT TILs were detected. Maximal numbers of IT TILs at the IM per 20× objective FOV fluctuated between 0 and 40% (MV: 1%; SD: 4%). The minimal numbers of IT TILs per 20× objective FOV extended from 0–2% (MV: 0.07%; SD: 0.32%).

### 3.3. Lymphocytes and Plasma Cells in the Adjacent Non-Neoplastic Tissue

Non-neoplastic mammary tissue was included in 100 of 107 sections of rabbit mammary carcinomas. Average percentages of lymphocytes and plasma cells within the inter- and intralobular fibrous connective tissue differed from 0–2% (MV: 0.11%; SD: 0.35%). Within the fibrous connective tissue, maximal percentages of lymphocytes and plasma cells per 20× objective FOV ranged from 0–5% (MV: 1%; SD: 0.52%) and minimal percentages of lymphocytes and plasma cells per 20× objective FOV from 0–2% (MV: 0.04%; SD: 0.24%). Examined cases did not show evidence of lobulitis.

### 3.4. Histological and Immunohistochemical Features

In the rabbit mammary carcinomas, the percentage of tubular growth in relation to the entire tumor area varied between 5% and 90% with a mean of 56% and a SD of 25% (Table 2). Vast majority of tumors (97%; 104/107) showed moderate cellular pleomorphism, whereas only 2% and 1% had mild and marked cellular pleomorphism, respectively. The numbers of mitotic figures per ten 40× HPFs ranged from 0–32 mitoses with a MV of six mitotic figures and a SD of six mitoses (Table 2).

**Figure 4:**
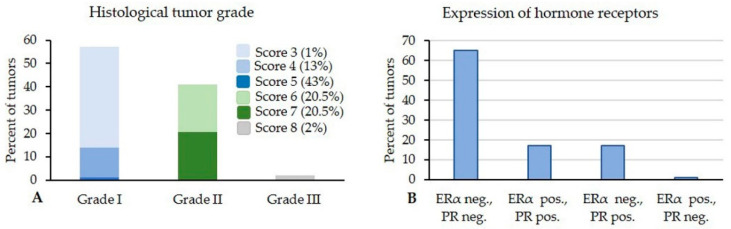
(**A**) Of the 107 rabbit mammary carcinomas, 57% were grade I, 41% grade II, and 2% grade III. (**B**) The majority (65%) was hormone receptor negative, whereas 17% of the tumors expressed both receptors, 17% showed immunostaining for solely progesterone receptor, and 1% was immunoreactive for only estrogen receptor. ERα = estrogen receptor; PR = progesterone receptor.

Well differentiated grade I tumors predominated with 57% (61/107); the frequency of the grading scores 3, 4, and 5 was 1%, 13%, and 43%, respectively. Moderately differentiated grade II tumors occurred with 41% (44/107); 20.5% were consisted with grading score 6 and 20.5% with grading score 7, respectively. Poorly differentiated grade III tumors were observed with 2% (2/107), all of these were score 8 (Figure 4).

**Figure 5:**
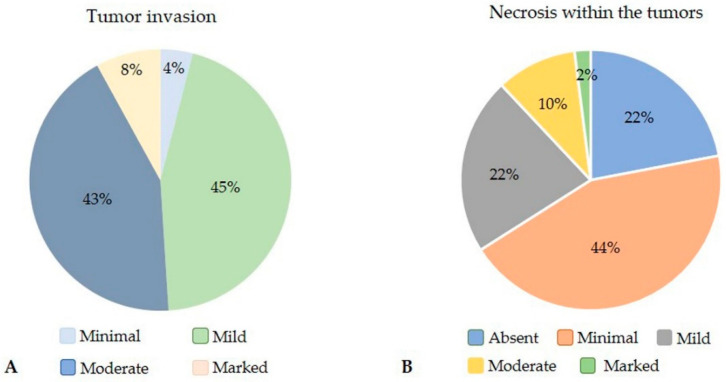
(**A**) Rabbit mammary tumors showed mostly mild (45%) and moderate (43%) invasive behavior; only small percentages of tumors displayed minimal (4%) and marked (8%) tissue invasion. (**B**) Necrosis was absent in 22% of the tumors, whereas minimal and mild necrosis was detected in 44% and 22% of rabbit mammary carcinomas, respectively. Moderate necrosis was present in 10% of the tumors and 2% of tumors had marked necrosis.

The degree of invasion could not be evaluated in four carcinomas (4%) because of incomplete excision of the tumor. Peritumoral tissue was present in 103 carcinomas. Of these, four carcinomas (4%; 4/103) were well demarcated with multifocal only minimal invasion in the peritumoral tissue, whereas 45% (46/103), 43% (45/103), and 8% (8/103) displayed mild, moderate, and marked invasion (Figure 5).

Areas of necrosis were absent in 22% of the carcinomas (23/107). Minimal necrosis that was detected in 44% of the tumors (47/107) was more frequently observed than mild, moderate, and marked necrosis that occurred in 22% (24/107), 10% (11/107), and 2% (2/107) of the carcinomas, respectively (Figure 5).

Immunostaining of the carcinomas showed that 81% (87/107) were negative for ERα (IRS and H-score: 0) and 66% (71/107) for PR (IRS and H-score: 0). In detail, 65% of the carcinomas (70/107) lacked expression of both receptors, 17% (18/107) were double positive for ERα and PR, 17% (18/107) were solely positive for PR, and 1 tumor was only positive for ERα (Figure 4). The positive IRS for ERα varied between 0.15 and 2.28, whereas the positive IRS for PR ranged from 0.16 to 3.66. The positive H-score for ERα ranged from 14.87 to 101.35, and the positive H-score for PR differed between 9.30 and 149.07 (Table 2).

The percentage of calponin positive tumor cells showed a variation between 0% and 22% with a MV of 8% and a SD of 5% (Table 2). In detail, 7% of the carcinomas (8/107) lacked calponin expression in tumor cells, whereas 65% (69/107) contained ≤ 10% calponin positive tumor cells and 28% (30/107) had 11–22% of calponin expressing neoplastic cells.

### 3.5. Statistical Correlations

The results of the statistical analyses are summarized in Table 3. A significant association existed between average stromal TILs within the CT and average stromal TILs at the IM. For both locations, average stromal TILs and maximal stromal TILs at 20× objective FOV were statistically correlated as well. In addition, at the IM a further correlation existed between average stromal TILs and average IT TILs.

**Figure 6:**
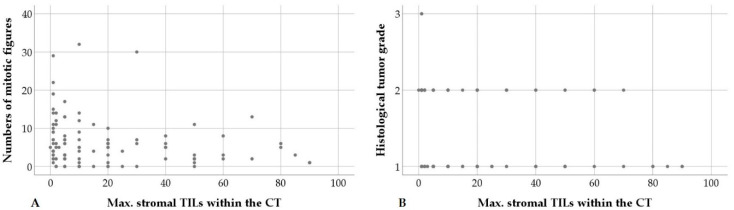
Higher maximal (max.) stromal tumor infiltrating lymphocytes (TILs) per 20× objective field of view (FOV) within the central tumor (CT) are statistically correlated with decreased numbers of mitotic figures per ten 40× HPFs (**A**) and a lower histological tumor grade (**B**).

Higher maximal numbers of stromal TILs in the CT showed a statistical association with a decreased mitotic count, as well as a lower grading score and tumor grade (Figure 6).

**Figure 7:**
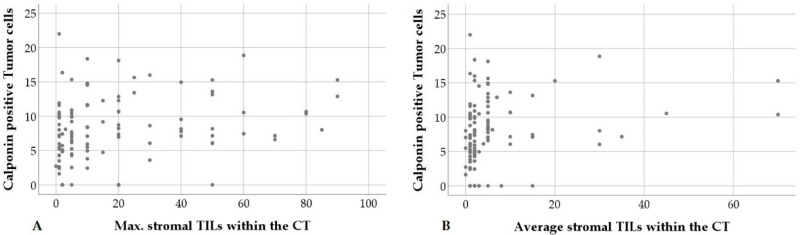
An increased percentage of calponin positive tumor cells is statistically correlated with higher maximal (max.) stromal tumor infiltrating lymphocytes (TILs) per 20× objective FOV within the central tumor (CT), (**A**) and higher average stromal TILs within the central tumor (**B**).

No statistical association was observed between evaluated TIL parameters and the ERα/PR status. An increased percentage of calponin positive tumor cells, however, was correlated with higher maximal numbers of stromal TILs within the CT as well as higher percentages of average stromal TILs within the CT and at the IM (Figure 7).

## 4. Discussion

To the best of the authors knowledge this is the first study to examine TILs in mammary carcinomas of pet rabbits. For this, the international guidelines for TIL evaluation on human breast cancer were used [20,21,31], since only a limited number of studies on TILs in mammary carcinomas of different animal species have been published so far and standardized guidelines are not available yet. The adoption of the guidelines for TIL evaluation in human breast cancer to mammary tumors of pet rabbits and domestic animals has the advantage of not only allowing a standardized assessment across different animal species, but also between individual animal species and human beings.

### 4.1. Evaluation of Tumor Infiltrating Lymphocytes in Routinely Fixed, Processed, and Stained Tissue

The use of HE stained sections of FFPE tissue for TIL evaluation offers the following advantages for scientific studies on pet rabbits and other across animal species.

It can be readily included in the routine diagnostic examination of tissue sections [21] and allows retrospective studies on archived material. Moreover, it is a cost- and time-effective method, since it avoids additional tests requiring further laboratory work and equipment as well as further examinations at later time points [21]. Cost restriction can be an important aspect for veterinary medicine since pet owners may have financial limitations.

In addition, the assessment of TILs in HE stained specimens can be applied to human beings and all domestic and pet animals, since it does not include the need for subclassification of lymphocyte subpopulations and the resulting requirement for consideration of species-specific expression of lymphocyte markers. The immunohistochemical subclassification of lymphocyte subpopulations beyond T cells and B cells is often not established in the routine diagnostic work up of veterinary pathology laboratories.

### 4.2. Distribution of Stromal and Intra-Tumoral Tumor Infiltrating Lymphocytes

The present investigation showed a significant correlation between average and maximal stromal TILs presence at CT and IM suggesting an overall similar level of immune cell presence and presumptive activation across a given carcinoma.

However, the distribution of stromal TILs within the CT was often heterogenous, i.e., 47% of carcinomas showed a difference of >10% between maximal and minimal numbers of stromal TILs per 20× objective FOV. It can be speculated that the TIL hotspots may be observed in areas containing tumor cell clones expressing or having expressed immunogenic antigens [37]. Spatial heterogeneity of TILs also occurs in human breast cancer [20,21,37,38].

At the IM of the rabbit mammary carcinomas, stromal TILs were often intimately associated with nests of invasive tumor cells. Since tumor invasion is associated with loss cohesiveness between carcinoma cells, it likely will facilitate penetration of lymphocytes in tumor cell nests. This assumption is supported by the finding that at the IM a statistically significant correlation existed between average numbers of stromal and IT TILs. In this regard, Salgado et al. [20] reflected that the distinction between IT TILs and stromal TILs may be arbitrary, since TILs are able to move between the different tumor compartments. After their migration in nests of tumor cells, TILs may, however, become entrapped there because of their ability to establish cell to cell contacts with tumor cells, i.e., TILs may express the integrin CD103 that binds to E-cadherin on cancer cells [39].

Notably, Nawaz et al. [38] revealed that spatial heterogeneity of immune cell infiltrates has prognostic significance in ERα negative breast cancer. They showed that a higher number of co-localized immune cell and cancer cell hotspots weighted by the tumor area correspond to a longer time for local and distant metastases [38].

### 4.3. Tumor Infiltrating Lymphocytes and Histological Features

There was a statistically significant association between increased maximal percentages of stromal TILs per 20× objective FOV and histological features indicative of a better tumor differentiation, i.e., reduced mitotic count, as well as lower grading scores and tumor grades. These data suggest that rabbit mammary carcinomas with a better differentiation are composed of immunogenic tumor cell clones that facilitate TIL accumulation [18,37]. Thus, it may be speculated that a higher percentage of maximal stromal TILs may represent a favorable prognostic factor in rabbit mammary carcinomas. This would support the assumption of Saltz et al. [37] that not only the average numbers of TILs, but also their spatial distribution has likely prognostic significance. As a next step, it would be interesting to analyze the spatial correlation of immune and cancer cell hotspots also in rabbit mammary carcinomas and to correlate these with microscopic features of tumor dignity.

### 4.4. Tumor Infiltrating Lymphocytes and Immunohistochemical Features

A statistically significant association existed between higher percentages of calponin positive tumor cells and increased average percentages of stromal TILs in the CT and at the IM as well as higher maximal numbers of stromal TILs in the CT per 20× objective FOV. The latter, as well as higher numbers of calponin positive tumor cells are significantly correlated with a lower mitotic count in neoplastic cells. These findings indicate that in pet rabbit mammary carcinomas both parameters, i.e., a higher number of calponin positive tumor cells, as well as an increased percentage of stromal TILs are associated with a better tumor differentiation and thus may have the potential to serve as biomarkers for a better tumor prognosis.

Calponin is linked to the actin cytoskeleton and acts as tumor suppressor protein by facilitating intercellular adhesion and inhibiting cellular motility and cell division [40,41]. By inhibiting mitotic activity of tumor cell [40,41], calponin may also help to prevent mutations in tumor cells that can lead to the development of less immunogenic subclones.

### 4.5. Tumor Infiltrating Lymphocytes in Tumors of Domestic Animals

In several tumors of animals, a prominent lymphocytic infiltration is associated with a favorable prognosis. For example, in canine cutaneous histiocytoma and oral papilloma it indicates tumor regression [42,43]. Similarly, in transmissible venereal sarcomas of dogs, the number of mononuclear immune cells was significantly higher in tumors that show regression or stable growth than in tumors with progression [44]. In rabbits with auricular VX2 carcinomas, tumors in remission contained statistically higher CD3 cell infiltrates than those exhibiting progression [6].

Only a few studies on TILs in mammary tumors of dogs are available [45,46,47,48,49]. In contrast to the present study, investigations in dogs revealed an association between a higher infiltration with TILs in mammary carcinomas and histological parameters indicative of a worse prognosis, i.e., a higher tumor grade [47], as well as the presence of lymphatic tumor cell emboli [47] or vascular invasion [48]. In addition, Estrela-Lima et al. [45] detected a statistically significant correlation between an intense lymphocytic infiltrate and a shorter survival. By distinguishing tumors with high and low CD3+ lymphocyte infiltrates, Saeki et al. [46] detected a significantly reduced survival rate within one year in the high CD3+ T cell infiltration tumor group. These studies examined TILs in different types of carcinomas including carcinoma in benign mixed tumor [45,47], complex carcinoma [47,48], simple carcinoma of different histotypes [45,47,48], squamous cell carcinoma [47], as well as carcinosarcoma [48] and used divergent evaluations [45,46,47,48]. Kim et al. [47] scored the lymphocytic infiltration based on their distribution as well as density, whereas Saeki et al. [46] and Carvalho et al. [48] counted CD3+ T cells within five and ten 40× HPFs, respectively. Estrela-Lima et al. [45] enumerated lymphocytes in eight “hot spots” fields.

In triple-negative complex type mammary carcinomas of dogs, not only total numbers of immune cells, but also their phenotype influenced survival [49]. In detail, statistically significant shorter survival times occurred in dogs with a total higher immune cell infiltrate as well as higher numbers of CD3+ T cells, CD4+ T cells or tumor infiltrating macrophages [49].

A comparison of these studies with each other, the present investigation, as well as data on human breast cancer is impaired by use of different methodical approaches. To the best of the authors knowledge, there are no published studies on mammary carcinomas and other tumors of dogs and cats and other pet animals that used the international guidelines for TIL evaluation on human breast cancer.

### 4.6. Tumor Infiltrating Lymphocytes in Human Breast Cancer

Majority of studies analyzed the association between TILs and clinical data of prognostic and predictive value, i.e., complete pathological response [50,51], disease free survival [27,51], or overall survival time [51] after certain treatment regimes. These investigations revealed that in certain types of breast cancer, in particular in HER2 positive and triple-negative types, increased stromal TILs are associated with a better clinical outcome [27,50,51] or can even predict response to certain treatment regimens [27,50]. Notably, the molecular signatures of tumor associated immune cells may further assist in patient stratification and treatment decisions [52]. In this regard, the immunological constant of rejection (ICR) analyzes the activation of genes associated with a cytotoxic immune response, Th1 signaling interferon and Th1 chemoattraction [52]. Within the groups of HER2 positive and triple- negative breast cancer, that by classical prognostic signatures are collectively defined as more aggressive breast cancer types, it serves as independent prognostic and predictive factor and can identify tumors with a presumable better prognosis and/or improved treatment response [52].

Data on a possible association of TILs and the histological tumor differentiation are only available in some publications. Several authors report higher average percentages of stromal TILs in breast cancer with higher histological grades [27,53] and/or a higher proliferation index [53].

These results cannot directly be compared with the present investigations, since in the rabbit mammary carcinomas a statistically significant correlation existed between the maximal percentage of stromal TILs and a lower histological grade. The maximal number of stromal TILs per 20× objective FOV were not included in the studies of Criscitiello et al. [53] and Loi et al. [27]. The study of Nawaz et al. [38] on human breast cancer, however, showed that the spatial heterogeneity of TILs and especially the colocalization of immune cell and cancer cell hotspots has prognostic significance.

As a possible explanation for the favorable prognostic value of TILs in human breast cancer, despite their possible association with a higher histological tumor grade [27,53] or higher proliferation index [53], might be related to their treatment-associated activation [17,20,50].

### 4.7. Future Perspectives on the Concept of “One Health, One Medicine”

#### 4.7.1. Standardized Evaluation of Tumor Infiltrating Lymphocytes

For a direct comparison of data between different animal species and human beings, standardized evaluation schemes are an essential diagnostic tool. This study shows that the international guidelines for TIL evaluation of human breast cancer [20,21,31] can be used for TIL assessment in mammary carcinomas of pet rabbits as well. Further, it provides evidence, that they can be applied also to mammary tumors of other domestic animals. Such a standardized evaluation approach will not only help to further characterize the value of TILs as prognostic and predictive biomarker in mammary carcinomas of different animal species, but it will also assist to find appropriate animal models for certain types of breast cancer in women. In addition, it may foster the development of effective anticancer immune-mediated therapies in veterinary and human medicine.

#### 4.7.2. Rabbits as Animal Model for Immunoncological Studies

Rabbits with experimentally induced VX2 carcinomas have been proposed as animal models for different types of human carcinomas including breast cancer [5,6,7]. Since VX2 mammary carcinomas do not show the molecular signatures defining different types of human breast cancer, they are not suitable to investigate specific molecular aspects of breast cancer tumorigenesis and immunoediting.

This study reveals that in mammary carcinomas of pet rabbits the TIL-associated immunological response shows similar morphological features as in human breast cancer. In addition, the immune systems of rabbits and humans contain similar lymphocytic subpopulations [1,54], and they are more closely related than those of rodents and humans [4,54]. Further, investigations on spontaneous mammary tumors in rabbits reflect more closely the interaction between immune system and breast cancer in humans than studies on rodent models with induced or transplanted tumors. Nowadays, rabbits are widely available and represent very popular companion animals in many countries of Europe and Asia, in USA and Australia [55]. In addition, pet rabbits are often exposed to similar environmental antigens than their owners.

For similar reasons, a canine breast cancer model has been propagated as well [56]. In contrast to rabbits and humans, canine mammary carcinomas often contain proliferated interstitial myoepithelial cells and non-neoplastic bone or cartilage [34].

Since majority of rabbit mammary carcinomas are ERα and PR negative, the rabbit has been proposed as a potential animal model for ERα/PR negative breast cancer in humans [10] as well as potentially also for triple negative human breast carcinomas [12]. In addition, rabbits may be a suitable model for breast cancer types with a myoepithelial differentiation of tumor cells and a prolactin-associated tumorigenesis as well [10,11,12,15].

## 5. Conclusions

To the best of the authors knowledge, this is the first study that characterizes TILs in pet rabbit mammary carcinomas. It shows that the international guidelines for TIL evaluation in human breast cancer can be applied to mammary carcinomas of pet rabbits as well. The obtained data suggest that the evaluation of stromal TILs in rabbit mammary carcinomas should not only include average numbers but also hotspot areas.

## Figures and Tables

**Table 1:** Percentages of stromal and intra-tumoral tumor infiltrating lymphocytes.

TIL Numbers	Stromal TILs in CT (%)	Stromal TILs at IM (%)	IT TILs in CT (%)	IT TILs at IM (%)
**Average**				
Range	0.00–70.00	0.00–30.00	0.00–5.00	0.00–10.00
Mean ± SD	6.21 ± 11.61	3.34 ± 5.23	0.24 ± 0.67	0.24 ± 1.07
**Max. per 20×**				
Range	1.00–90.00	0.00–70.00	0.00–25.00	0.00–40.00
Mean ± SD	19.20 ± 23.29	10.28 ± 13.32	0.98 ± 3.00	1.00 ± 4.00
**Min. per 20×**				
Range	0.00–60.00	0.00–10.00	0.00–1.00	0.00–2.00
Mean ± SD	2.09 ± 6.97	1.11 ± 1.65	0.06 ± 0.23	0.07 ± 0.32

CT = central tumor; IM = invasive margin; IT = intra-tumoral; Max. = maximal numbers; Min. = minimal numbers; SD = standard deviation; TILs = tumor infiltrating lymphocytes; 20× = 20× objective field of view.

**Table 2:** Results of histological and immunohistochemical evaluations.

Analyzed Parameters	Range	Mean ± SD
Tubular growth	5–90%	56% ± 25%
Mitoses per ten 40× HPFs	0–32	6 ± 6
IRS estrogen receptor	0–2.28	0.16 ± 0.42
IRS progesterone receptor	0–3.66	0.56 ± 1.06
H-score estrogen receptor	0–101.35	11.29 ± 26.86
H-score progesterone receptor	0–149.07	25.44 ± 45.19
Calponin positive tumor cells	0–22%	8% ± 5%

HPFs = high power fields; H-score = histological score; IRS = immunoreactive score; SD = standard deviation.

**Table 3:** Results of statistical correlations.

Parameter 1	Parameter 2	*p*-Value, ρ-Value	Cases
**Tumor Infiltrating Lymphoc Ytes**			
Average stromal TILs in CT	Max. stromal TILs in CT	*p* = 0.000; ρ = 0.731	*n* = 107
	Average stromal TILs at IM	*p* = 0.000; ρ = 0.563	*n* = 102
	Max. stromal TILs at IM	*p* = 0.000; ρ = 0.420	*n* = 102
Max. stromal TILs in CT	Max. stromal TILs at IM	*p* = 0.000; ρ = 0.546	*n* = 102
Average stromal TILs at IM	Max. stromal TILs at IM	*p* = 0.000; ρ = 0.698	*n* = 102
	Max. stromal TILs in CT	*p* = 0.000; ρ = 0.436	*n* = 102
	Average IT TILs at IM	*p* = 0.038; ρ=0.206	*n* = 102
Average IT TILs in CT	Max. IT TILs in CT	*p* = 0.000; ρ = 0.831	*n* = 107
	Max. IT TILs at IM	*p* = 0.000; ρ = 0.734	*n* = 102
	Average IT TILs at IM	*p* = 0.000; ρ = 0.739	*n* = 102
Max. IT TILs in CT	Max. IT TILs at IM	*p* = 0.000; ρ = 0.936	*n* = 102
	Average IT TILs at IM	*p* = 0.000; ρ = 0.899	*n* = 102
**Histological Features**			
Degree of invasion	Degree of necrosis	*p* = 0.000; ρ = 0.352	*n* = 103
Grading score	Degree of invasion	*p* = 0.000; ρ = 0.364	*n* = 103
	Degree of necrosis	*p* = 0.003; ρ = 0.284	*n* = 107
Tumor grade	Degree of invasion	*p* = 0.002; ρ = 0.299	*n* = 103
	Degree of necrosis	*p* = 0.029; ρ = 0.211	*n* = 107
**Tumor Infiltrating Lymphocytes and Histological Features**			
Max. stromal TILs in CT	Mitotic count	*p* = 0.042; ρ = −0.197	*n* = 107
	Grading score	*p* = 0.035; ρ = −0.204	*n* = 107
	Tumor grade	*p* = 0.027; ρ = −0.213	*n* = 107
**Immunohistochemical and Histological Features**			
IRS estrogen receptor	IRS progesterone receptor	*p* = 0.000; ρ = 0.606	*n* = 107
	Mitotic count	*p* = 0.012; ρ = −0.243	*n* = 107
IRS progesterone receptor	Mitotic count	*p* = 0.008; ρ = −0.255	*n* = 107
	Degree of necrosis	*p* = 0.009; ρ = −0.250	*n* = 107
Calponin pos. tumor cells	Mitotic count	*p* = 0.035; ρ = −0.204	*n* = 107
**Calponin Positive Tumor Cells and Tumor Infiltrating lymphocytes**			
Calponin positive tumor cells	Max. stromal TILs in CT	*p* = 0.012; ρ = 0.241	*n* = 107
	Average stromal TILs in CT	*p* = 0.026; ρ = 0.215	*n* = 107
	Average stromal TILs at IM	*p* = 0.026; ρ = 0.220	*n* = 107

CT = central tumor; IM = invasive margin; IRS = immunoreactive score; IT = intra-tumoral; Max. = maximal; Min. = minimal; TILs = tumor infiltrating lymphocytes; *n* = numbers of evaluated cases.
